# Supplementary material for: The IL‐6 signaling complex is a critical driver, negative prognostic factor, and therapeutic target in diffuse large B‐cell lymphoma
Source: EMBO Mol Med. 2019 Sep 12;11(10):e10576. doi: 10.15252/emmm.201910576 (PMC6783642; doi:10.15252/emmm.201910576)
Supplement: Supplementary file 1 — Expanded View Figures PDF [file EMMM-11-e10576-s001.pdf]

Expanded View Figures

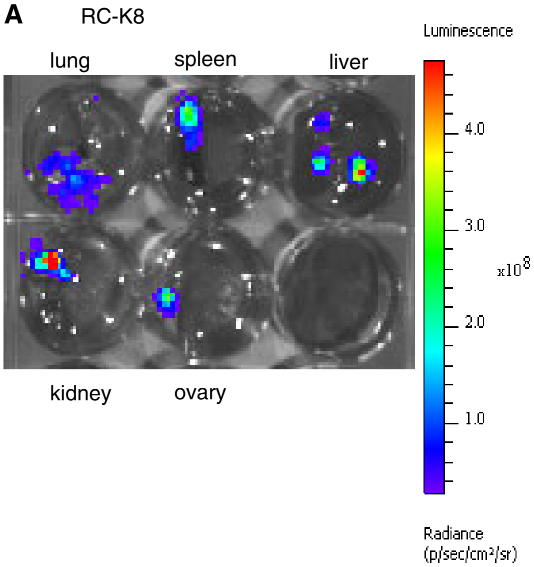

Figure EV1. DLBCL cells colonize various non-lymphoid tissues upon intravenous transplantation into MISTRG mice.

A total of 1×10^7 ZsGreen- and luciferase-expressing RC-K8 cells were intravenously injected into MISTRG mice and examined for tissue colonization at the study endpoint by IVIS. The color scale on the right indicates the radiance, i.e., the sum of the photons per second from each pixel inside the ROI/number of pixels (photons/s/cm^2/sr). The organs shown here were collected from one mouse, and are representative of all RC-K8-transplanted mice.

Figure EV2. Reconstitution of MISTRG mice with cord blood hematopoietic stem cells confers a growth advantage to xenotransplanted DLBCL cell lines in ectopic and orthotopic tumor models.

A Gating strategy for tumor cells and human immune cell subsets in reconstituted MISTRG mice: Tumor cells are gated as singlets/live/hCD45$^+$/hCD19-20$^+$/ZsGreen$^+$, whereas the other human immune subsets are gated on singlets/live/hCD45$^+$ and CD19/20$^+$ as B cells, CD3$^+$ as T cells, CD33$^+$ as myeloid cells, and NKp46$^+$ as NK cells. The equations used for calculating tumor burden and reconstitution efficiency are also shown. For assessing tumor burden in the spleen, tumor cells were gated as singlets/live/hCD45$^+$/ZsGreen$^+$, while those in the BM were additionally distinguished from non-malignant human immune cells using a B-cell marker (hCD19/20).

B A total of 1×10^7 U-2932 cells were subcutaneously transplanted into the flanks of 6-week-old reconstituted and un-reconstituted MISTRG mice. Median tumor volume and weight at the study endpoint (4 weeks) are shown. Each point on the plots represents the median tumor volume/weight of one experiment. Black symbols represent bulk U-2932, while red symbols are individual clones grown from sorted single cells.

C, D A total of 1×10^7 U-2932 cells were subcutaneously transplanted into the flanks of 6-week-old reconstituted MISTRG mice. Mice were treated with depleting antibodies against hCD4 or CCL2 starting on the day or tumor cell transplantation. Tumor volumes over time (presented as means ± SD), as well as tumor weight and tumor volume at the study endpoint, are shown. Each symbol represents one tumor.

E MISTRG mice were reconstituted at birth with 100,000–250,000 human CD34$^+$ cord blood HSPCs and intravenously injected with 1×10^7 U-2932 or RC-K8 cells at 6 weeks of age. The reconstitution efficiency (frequency of human among all CD45$^+$ leukocytes) in the bone marrow is shown for every mouse.

Data information: Horizontal lines indicate medians throughout.

 © 2019 The Authors

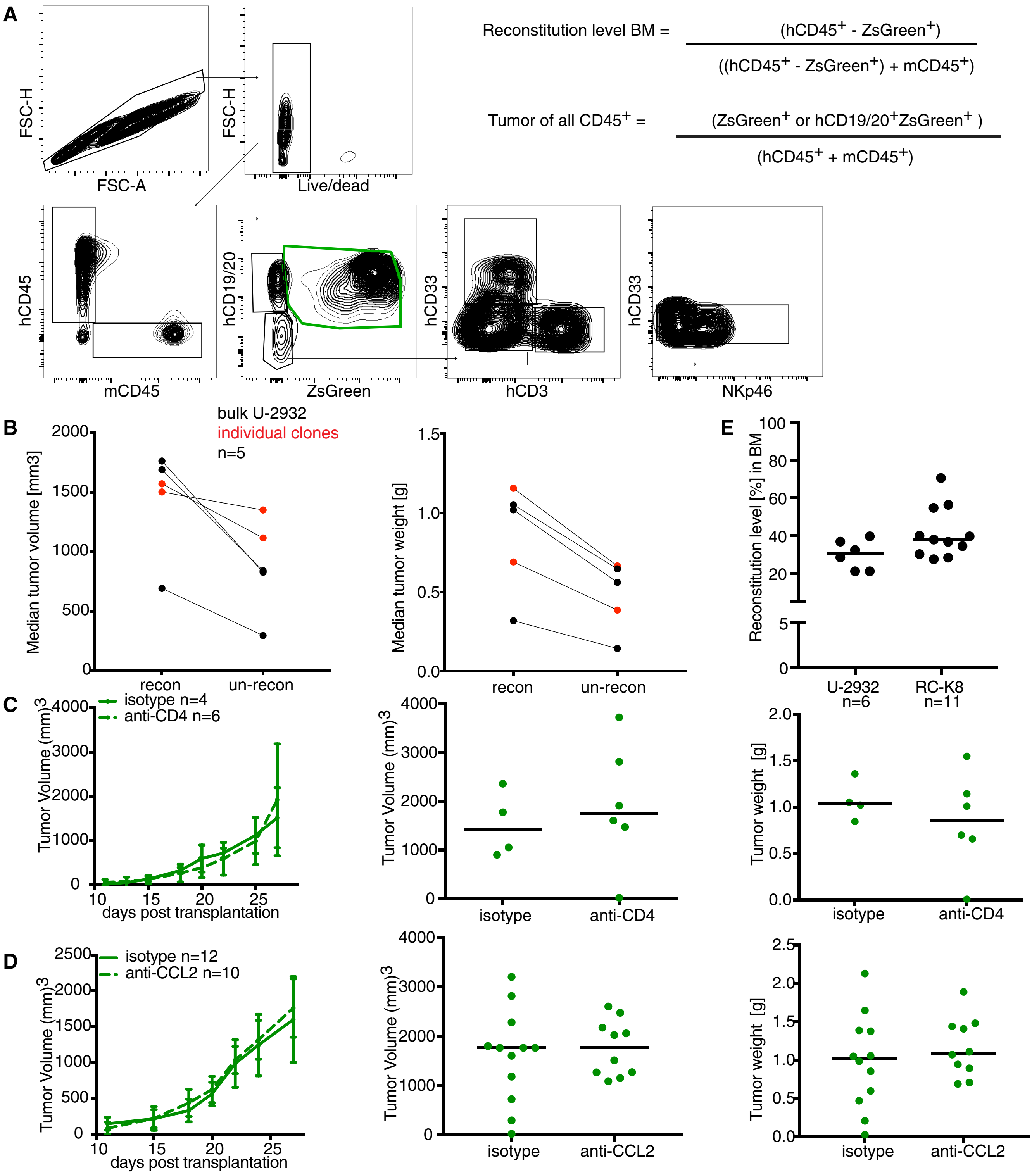
A
Reconstitution level BM = (hCD45+ - ZsGreen+) / ((hCD45+ - ZsGreen+) + mCD45+)
Tumor of all CD45+ = (ZsGreen+ or hCD19/20+ZsGreen+) / (hCD45+ + mCD45+)
FSC-H
FSC-A
Live/dead
hCD45
mCD45
hCD19/20
ZsGreen
hCD33
hCD3
NKp46
B
bulk U-2932
individual clones
n=5
Median tumor volume [mm3]
recon
un-recon
Median tumor weight [g]
E
Reconstitution level [%] in BM
U-2932 n=6
RC-K8 n=11
C
isotype n=4
anti-CD4 n=6
Tumor Volume (mm)3
days post transplantation
isotype
anti-CD4
Tumor weight [g]
D
isotype n=12
anti-CCL2 n=10
anti-CCL2

Figure EV2.

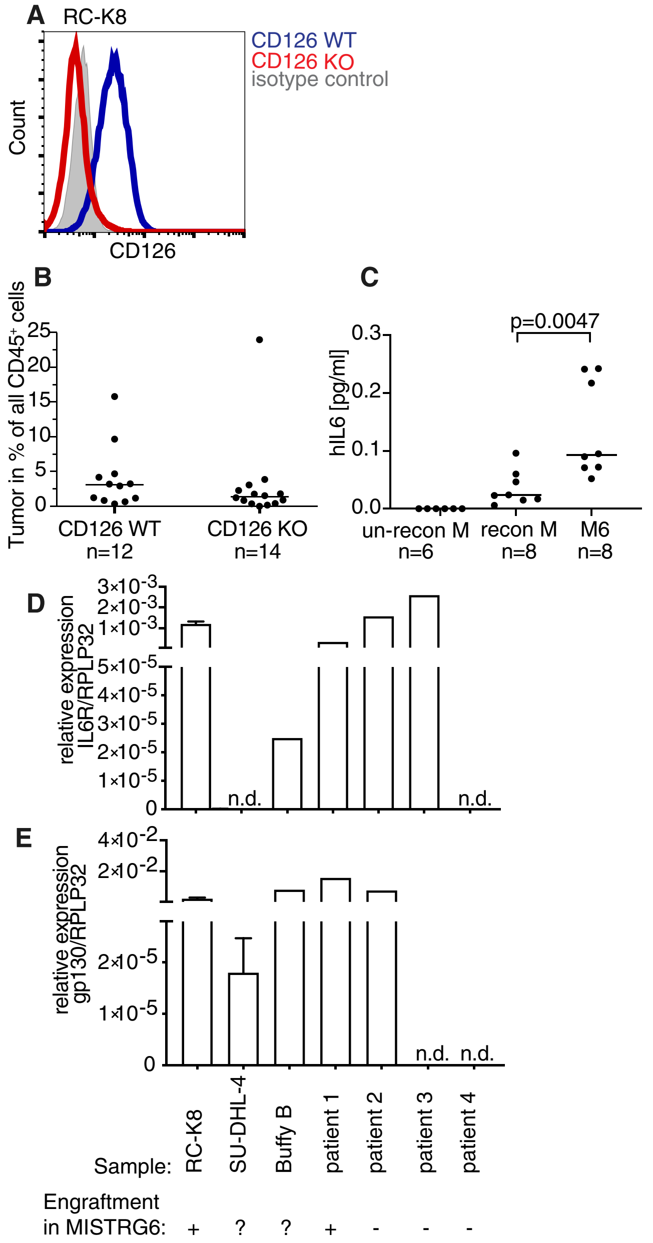

Figure EV3. The genetic ablation of CD126 expression reduces the tumor burden in MISTRG6 mice.

A RC-K8 cells were engineered to by CRISPR genomic editing for loss of CD126 expression. FACS histogram showing CD126 expression in WT and ko RC-K8 cells relative to isotype control antibody.

B MISTRG6 mice were intravenously injected with 1×10^7 CD126-proficient or CD126-deficient RC-K8 cells at 6 weeks of age; the lymphoma burden at the study endpoint (6 weeks p.i.) was quantified in the bone marrow by flow cytometric staining for hCD45 and by ZsGreen expression. Horizontal lines indicate medians. *P*-values were calculated using the Mann–Whitney test, but failed to reach statistical significance. Data are pooled from two independent studies. Each symbol represents one mouse.

C Human IL-6 levels in serum of MISTRG mice (reconstituted or not with human HSPCs for 10 weeks) and MISTRG6 mice, as determined by ELISA. Horizontal lines indicate medians, and each symbol represents one mouse. Mice were from two cohorts per group, but analyzed together on one ELISA plate. *P*-values were calculated using the Mann–Whitney test.

D, E qRT–PCR-based quantification of the IL-6R α-chain (D) and of the gp130 signaling chain (E) using RNA from immunomagnetically sorted $CD19^+$ patient-derived DLBCL cells (patients 1–4) relative to B cells sorted from buffy coats ("Buffy B") and a IL-6R-positive (RC-K8) and a IL-6R-negative (SU-DHL-4) DLBCL cell line. Whether the same sample engrafted in MISTRG6 within a time frame of 6 weeks after i.v. injection or not is indicated by + and − in the table below the graphs. ?, not tested. n.d., not detectable. qRT-PCR results of the cell lines are presented as mean ± SEM of two independent experiments. qRT-PCR results of patient 1–4 and Buffy B are single measurements.

 © 2019 The Authors

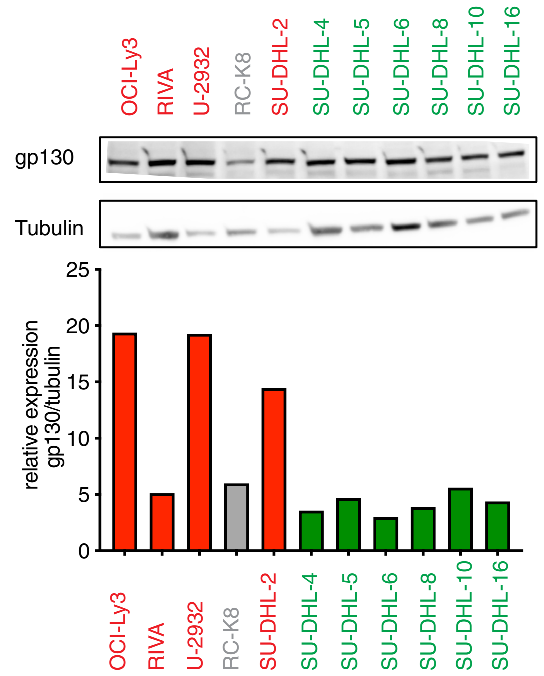

Figure EV4. gp130 expression is higher in the ABC than in the GCB subtype of DLBCL.

The expression of the IL-6R signaling chain gp130 was assessed by Western blotting performed on extracts from a panel of 11 DLBCL cell lines. ABC- and GCB-DLBCL cell lines are color-coded in red and green; RC-K8 is depicted in gray. The quantification of the blot relative to the tubulin loading control is shown in the lower panel. The Western blot is representative of two that were run with independently generated extracts.

Source data are available online for this figure.

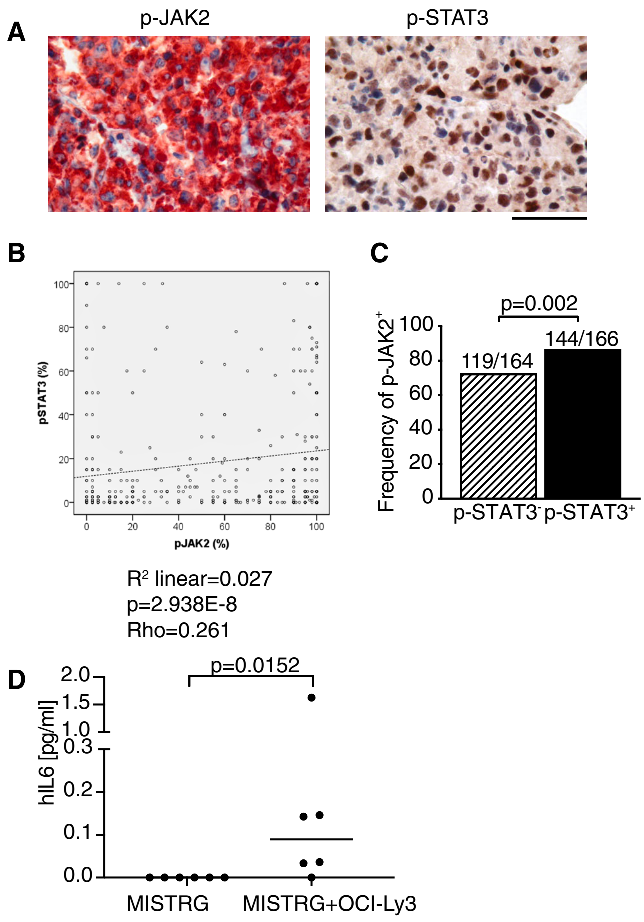

Figure EV5. STAT3 phosphorylation correlates with JAK2 phosphorylation in DLBCL.

A–C Biopsies of 330 cases of DLBCL were stained for p-JAK2 and p-STAT3 by immunohistochemistry, and the staining patterns of both antigens were correlated. A representative p-JAK2/p-STAT3-positive case is shown in (A), with p-JAK2 labeled in red and nuclear p-STAT3 labeled in brown (both counterstained with hematoxylin in blue). Frequencies of p-STAT3-positive cells among all tumor cells were plotted against p-JAK2 frequencies for all patients in (B), revealing a positive correlation between both markers. p-JAK2 positivity is more prevalent among p-STAT3-positive as opposed to p-STAT3-negative patients (C). The *P*-value in (C) was calculated using Fisher's exact test. Scale bar, 100 μm.

D Human IL-6 levels in serum of MISTRG mice that were transplanted or not with OCI-Ly3 cells and sacrificed after 4 weeks. Horizontal lines indicate medians, and each symbol represents one mouse. The *P*-value was calculated by the Mann–Whitney test.

Source data are available online for this figure.

© 2019 The Authors
