# Supplementary material for: The IL‐6 signaling complex is a critical driver, negative prognostic factor, and therapeutic target in diffuse large B‐cell lymphoma
Source: EMBO Mol Med. 2019 Sep 12;11(10):e10576. doi: 10.15252/emmm.201910576 (PMC6783642; doi:10.15252/emmm.201910576)

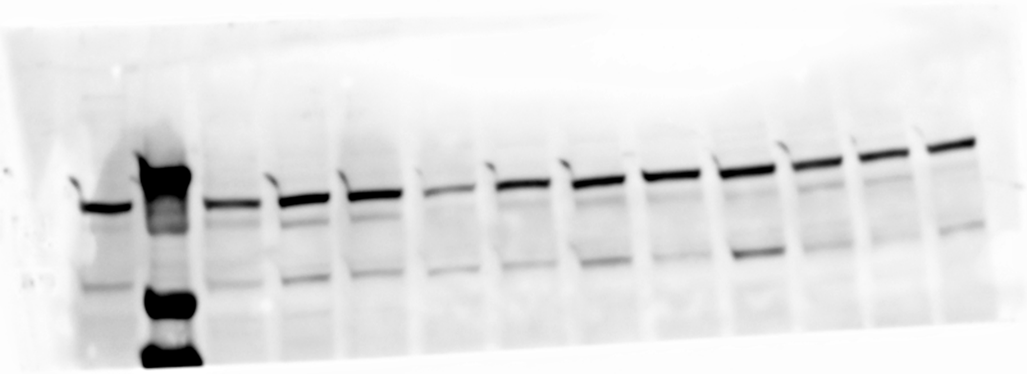

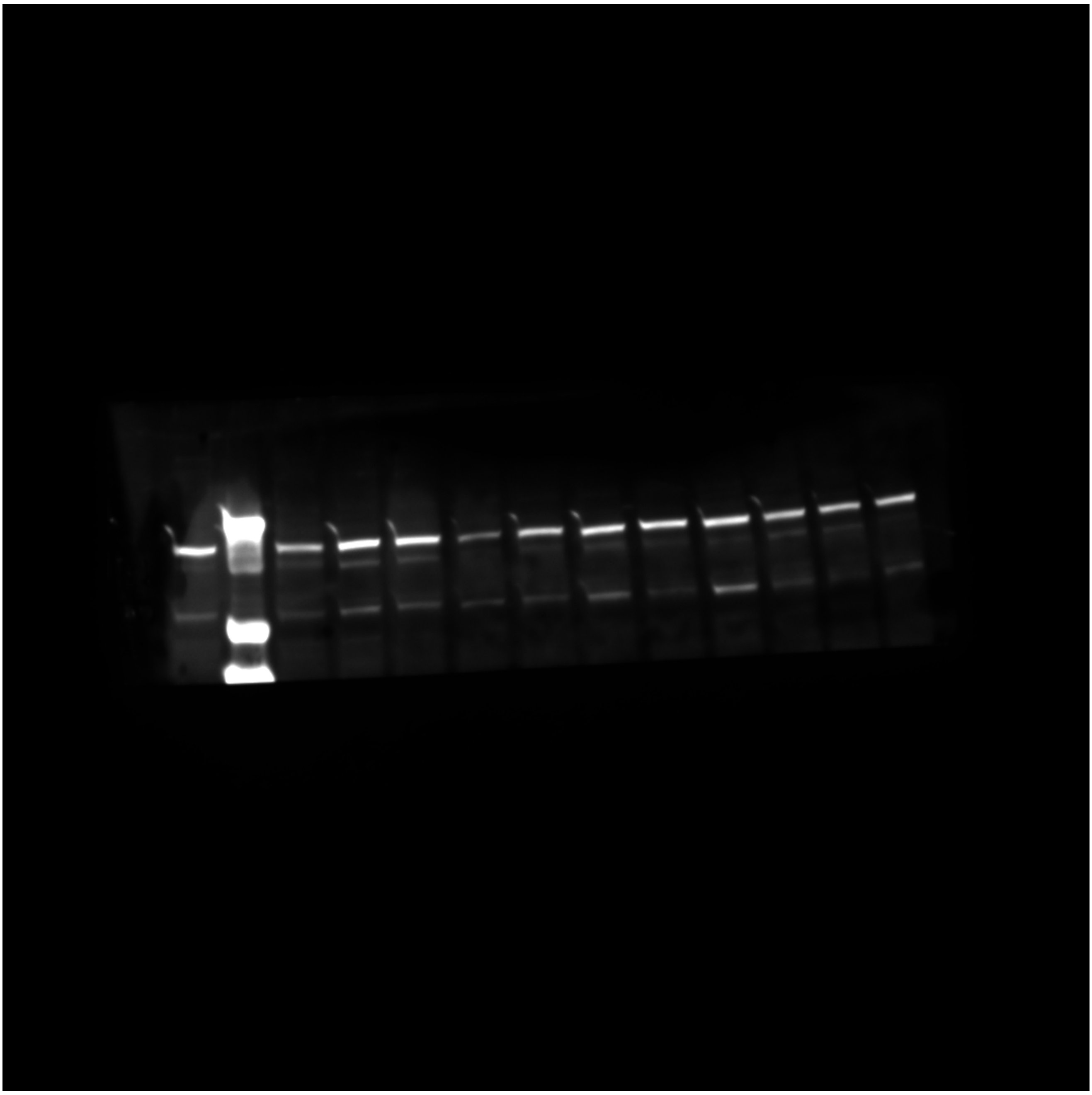

Supplement: Supplementary file 2 — Source Data for Expanded View [file EMMM-11-e10576-s006.zip › Source_Data_for_Expanded_View_Figures/WB_FigEV4A/Source_data_for_FigureEV4.pdf]
