# Supplementary figures and images for: The IL‐6 signaling complex is a critical driver, negative prognostic factor, and therapeutic target in diffuse large B‐cell lymphoma
Source: EMBO Mol Med. 2019 Sep 12;11(10):e10576. doi: 10.15252/emmm.201910576 (PMC6783642; doi:10.15252/emmm.201910576)

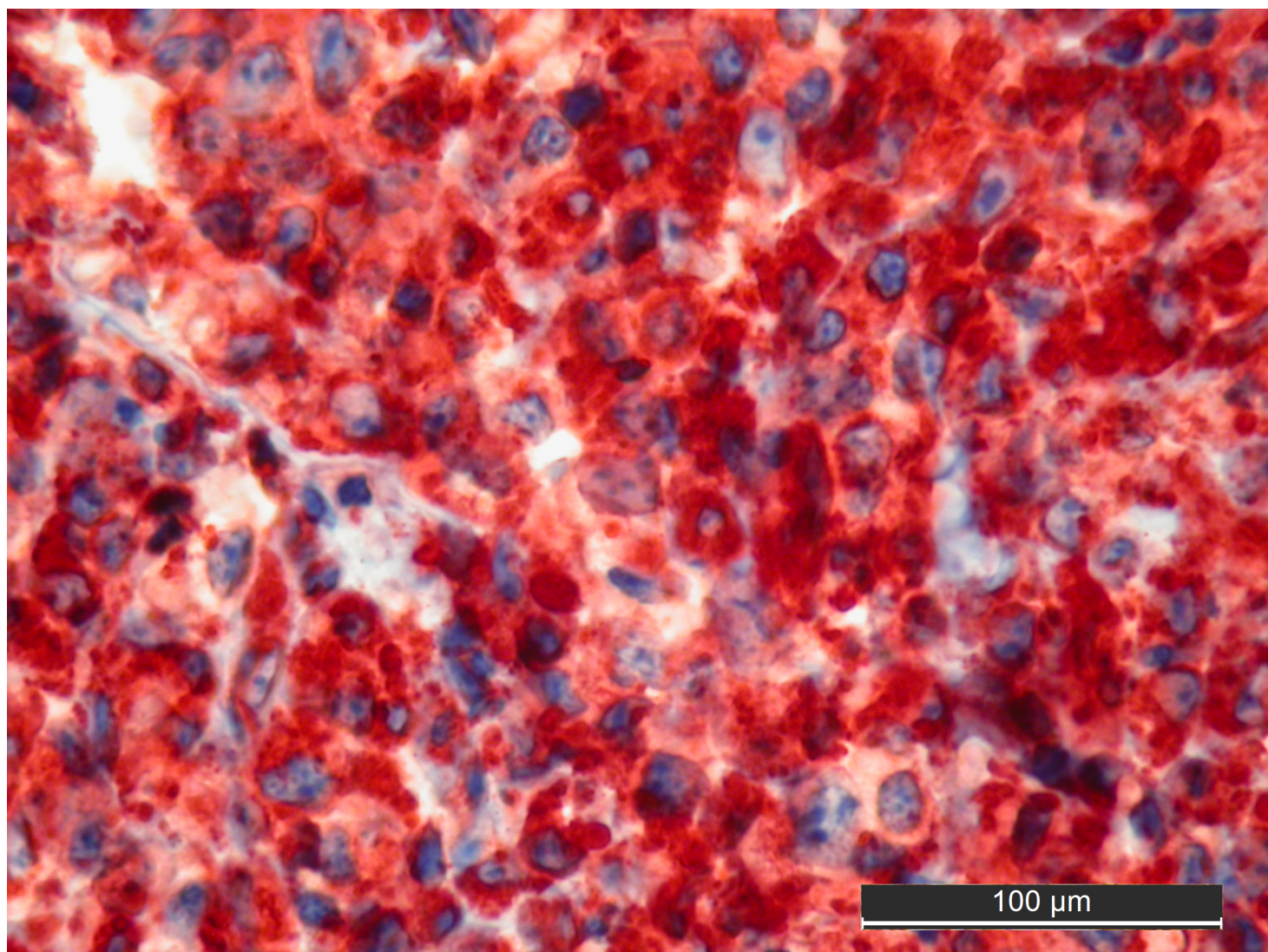

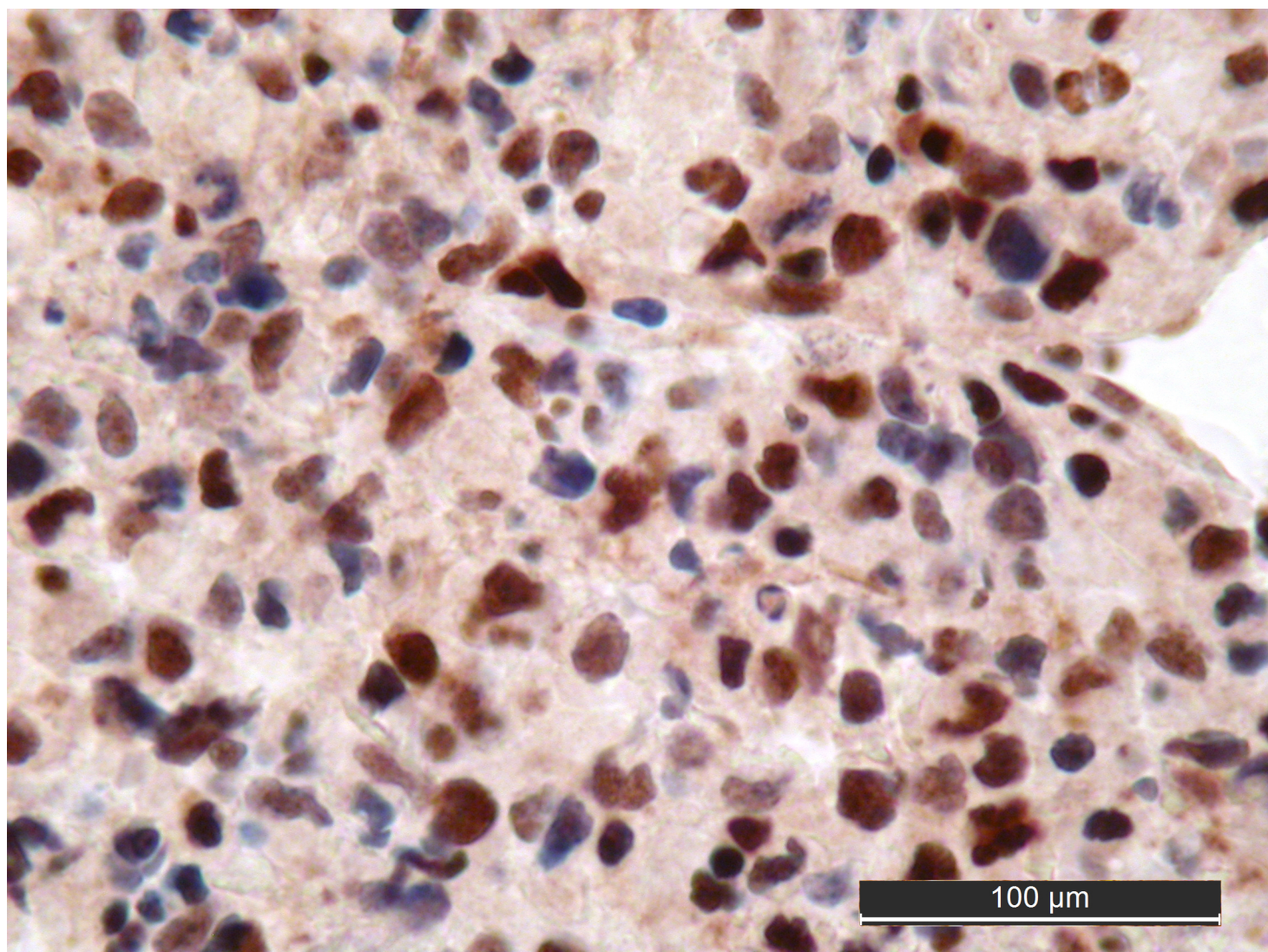

Supplement: Supplementary file 2 — Source Data for Expanded View [file EMMM-11-e10576-s006.zip › Source_Data_for_Expanded_View_Figures/IHC_FigEV5A/Source_data_for_FigEV5.pdf]

C

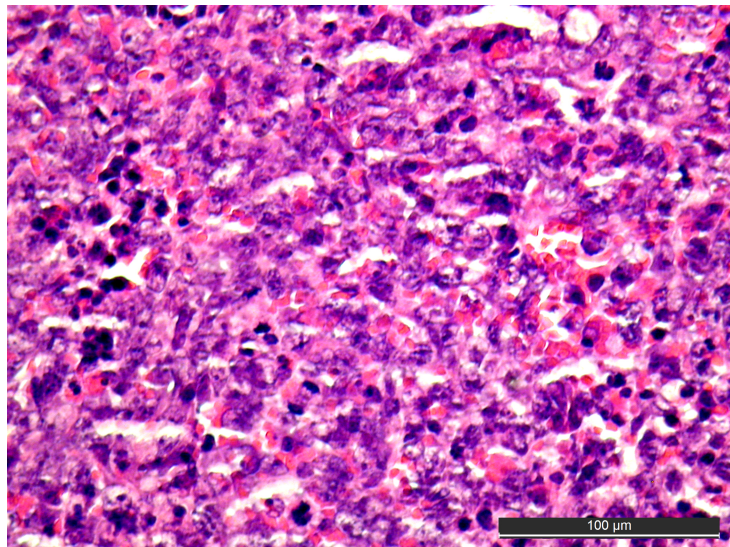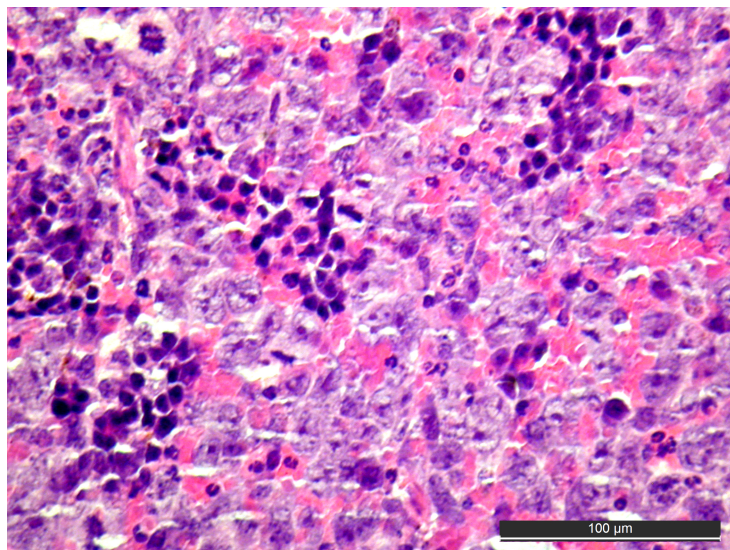

H

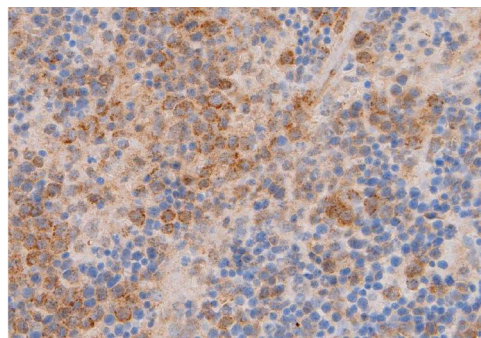

Supplement: Supplementary file 4 — Source Data for Figure 4 [file EMMM-11-e10576-s002.pdf]

**D**

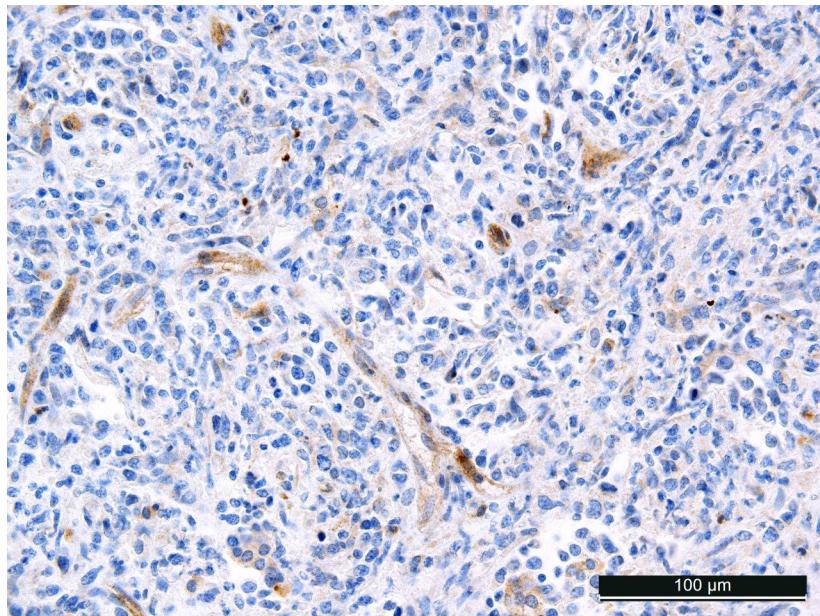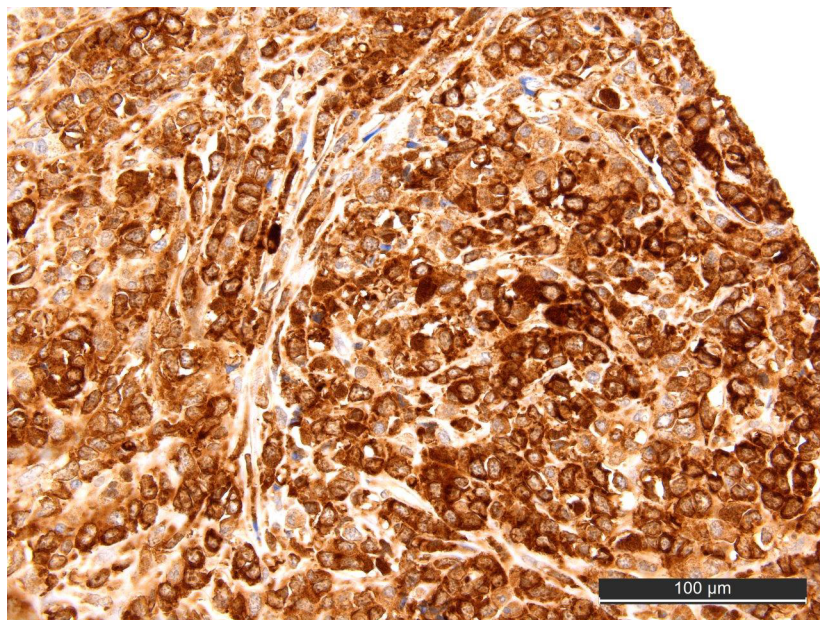

Supplement: Supplementary file 5 — Source Data for Figure 5 [file EMMM-11-e10576-s003.pdf]

**B**

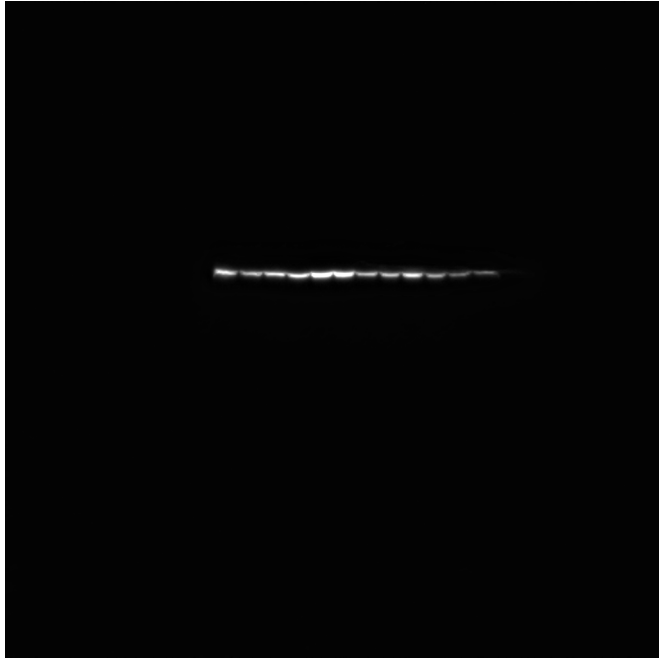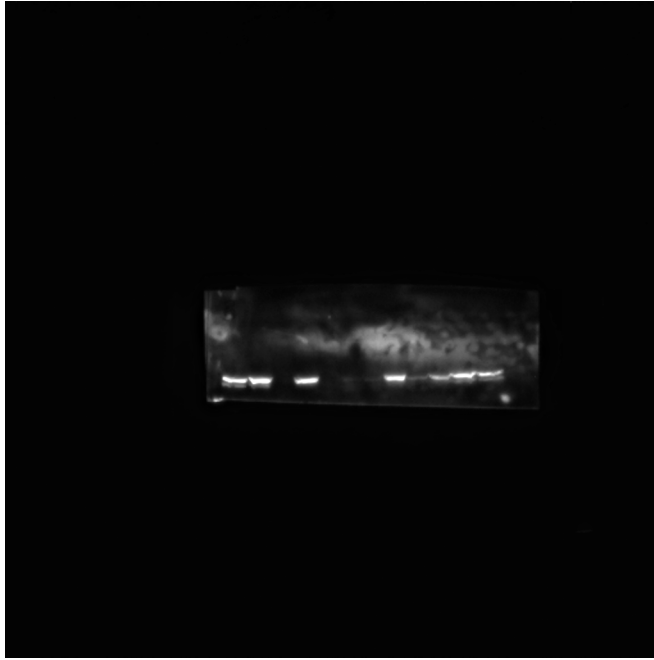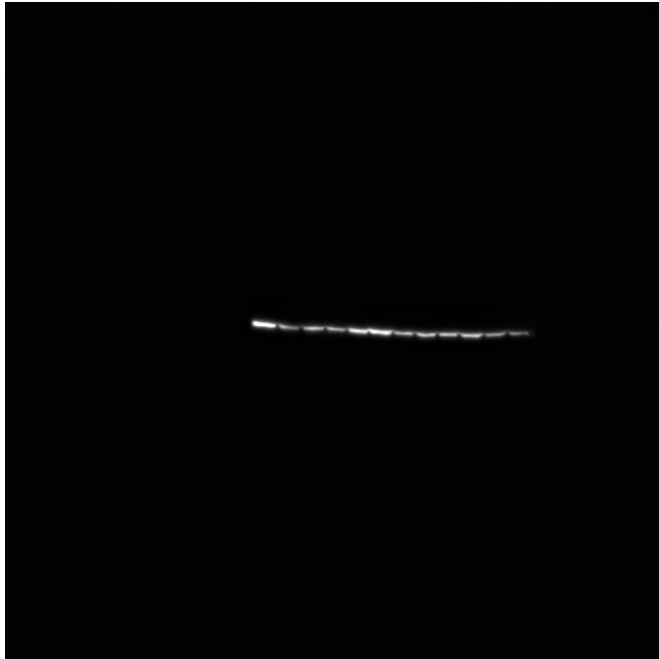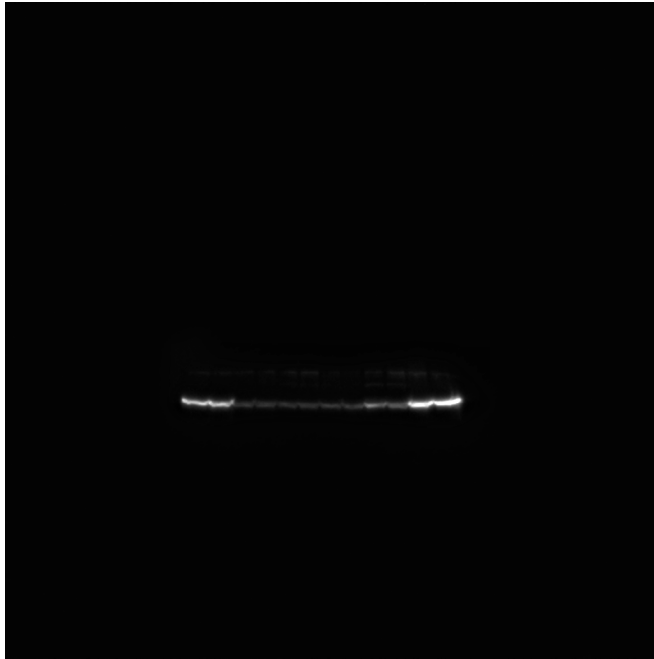

D

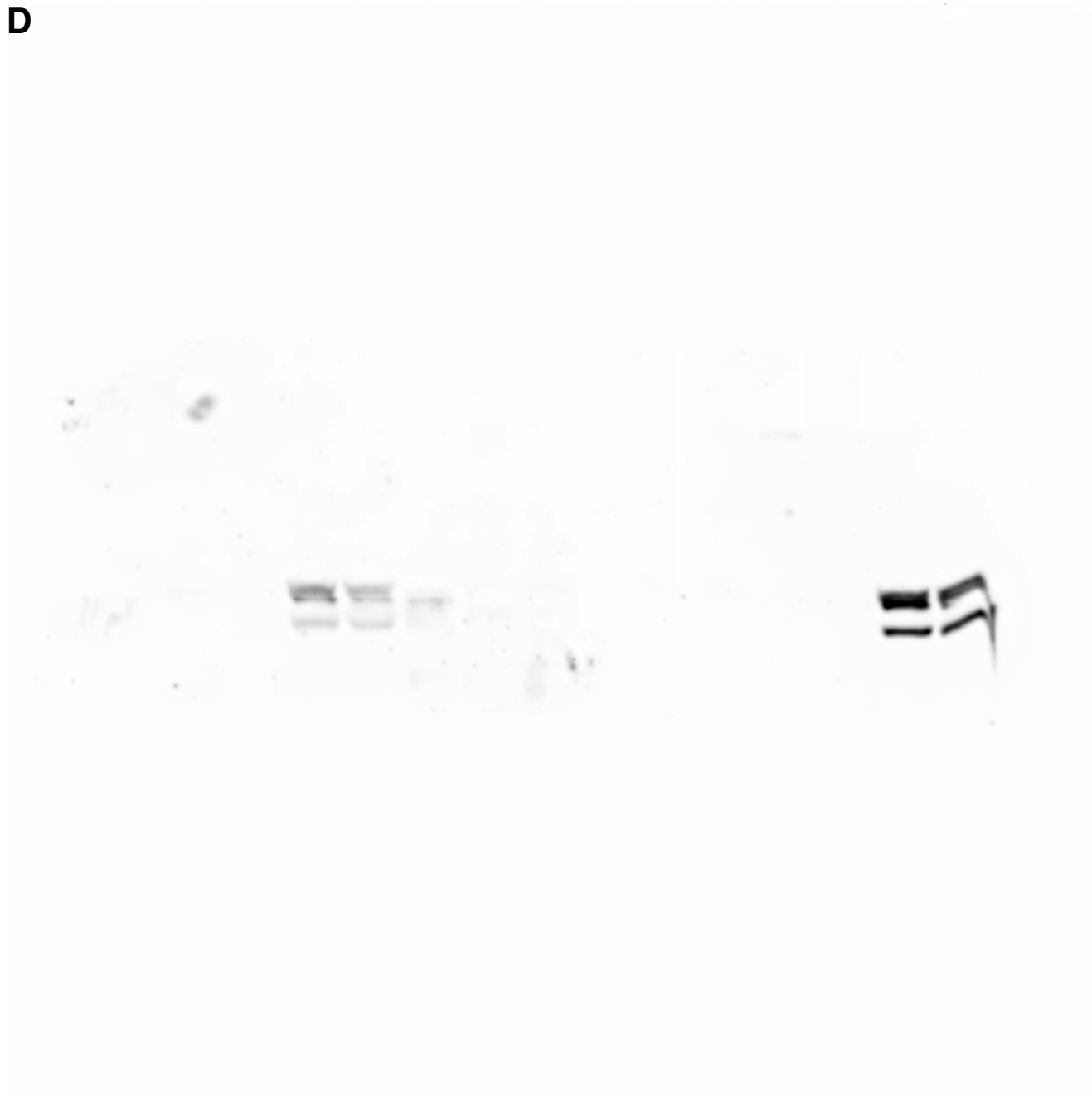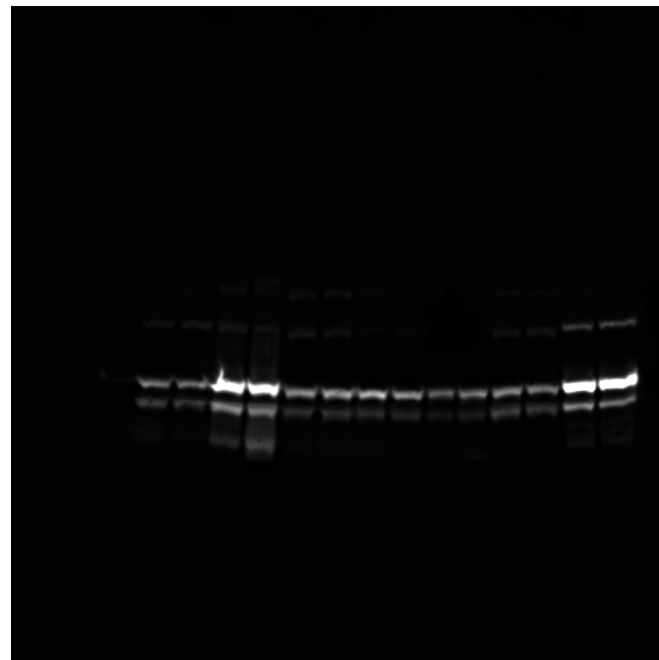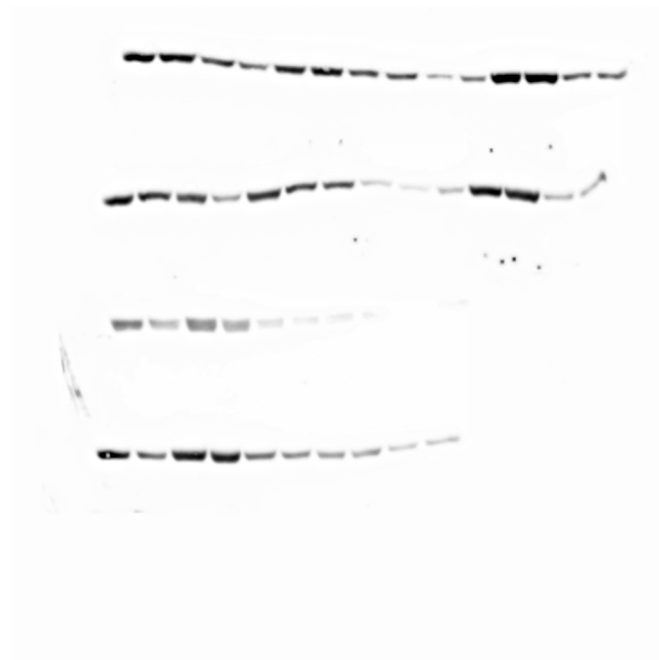

E

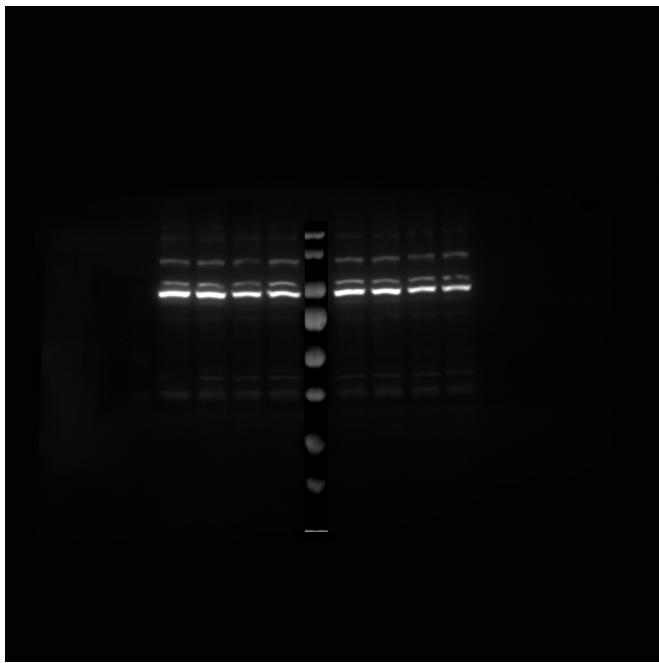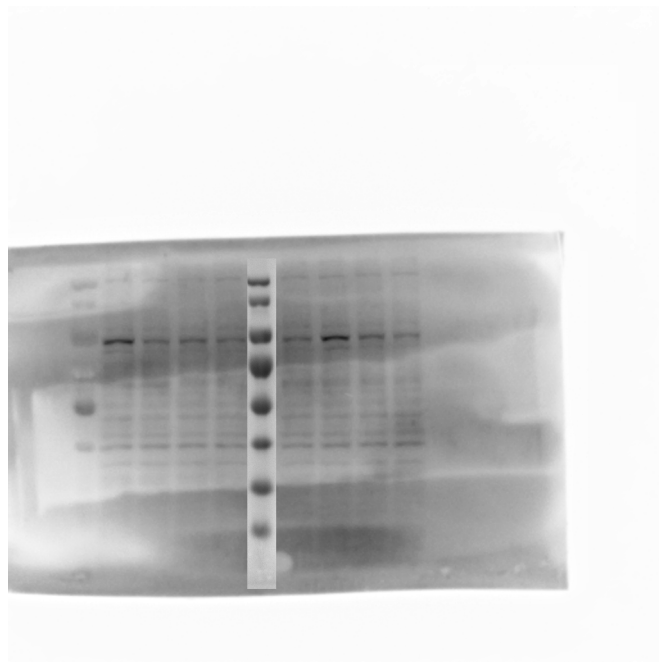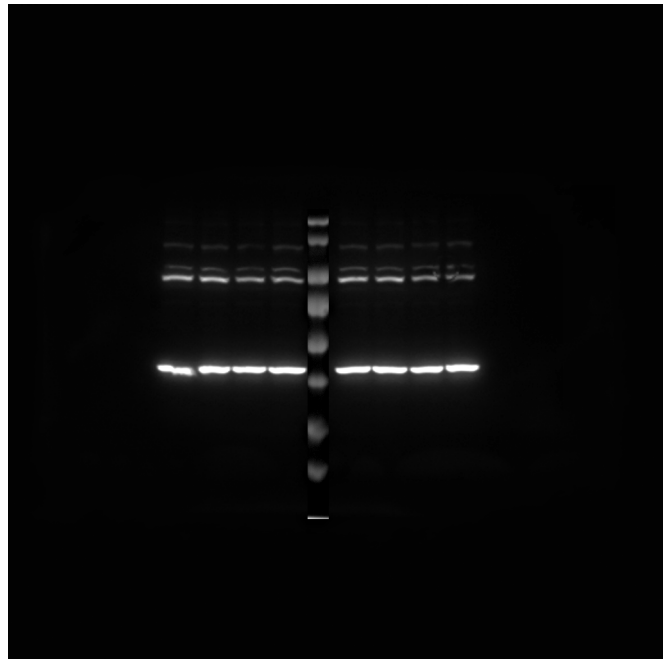

F

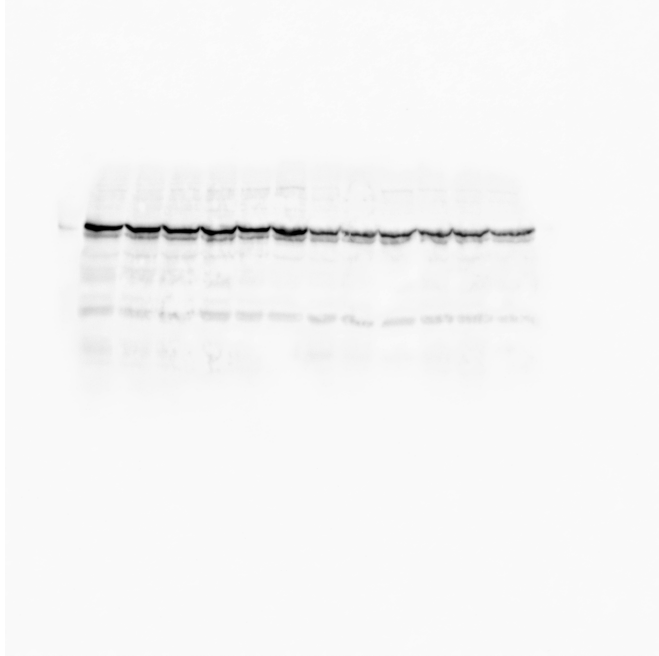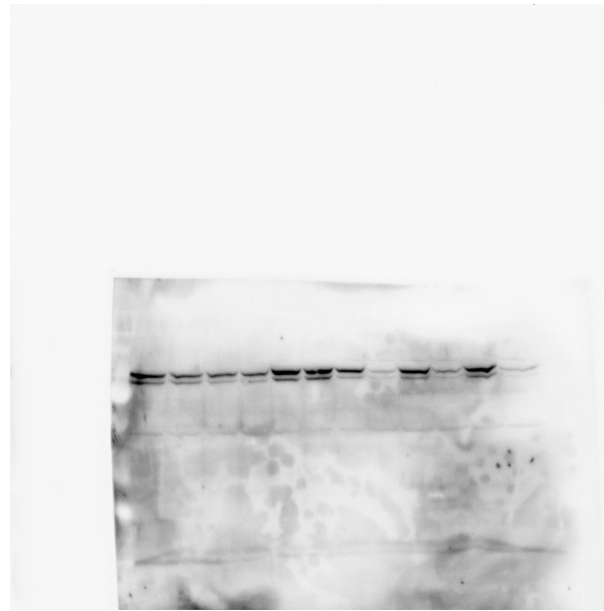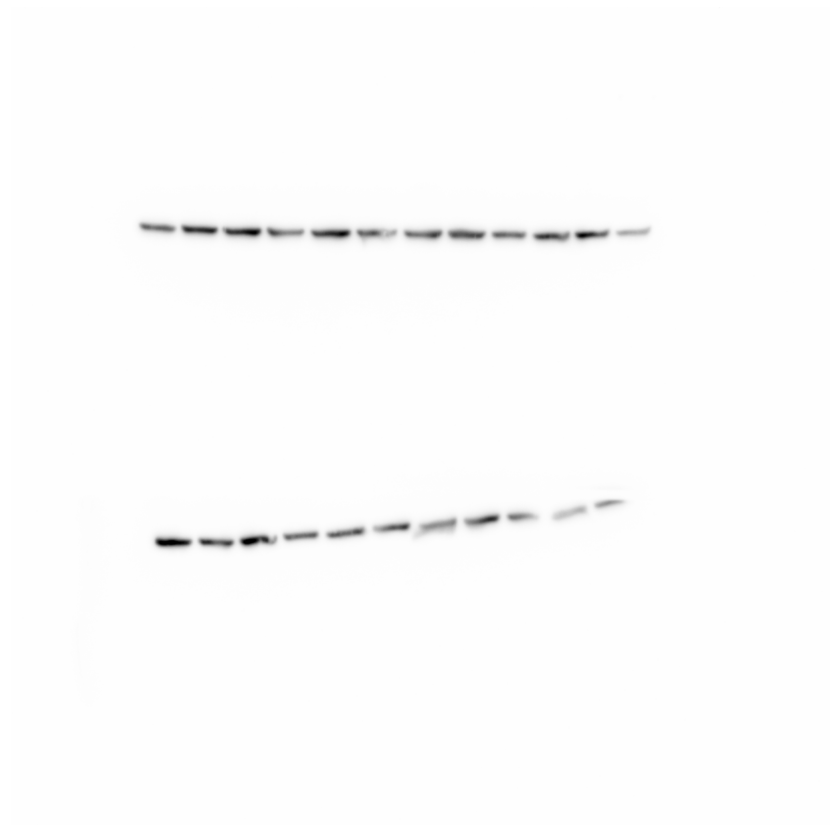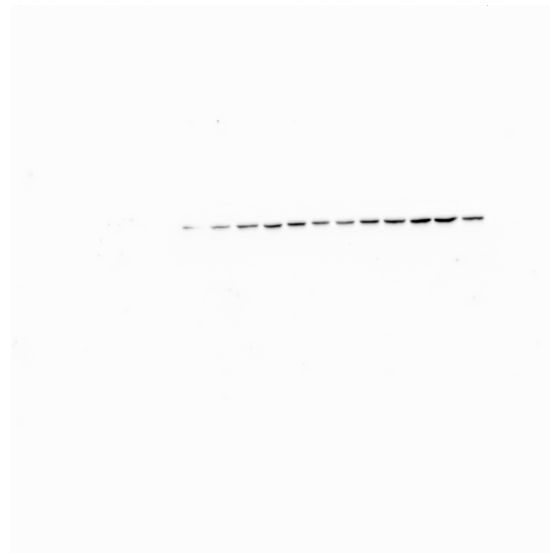

G

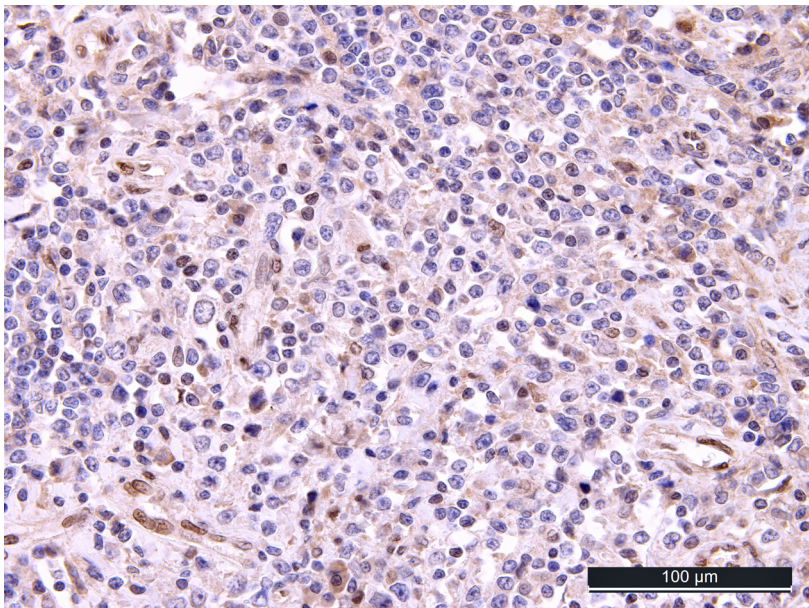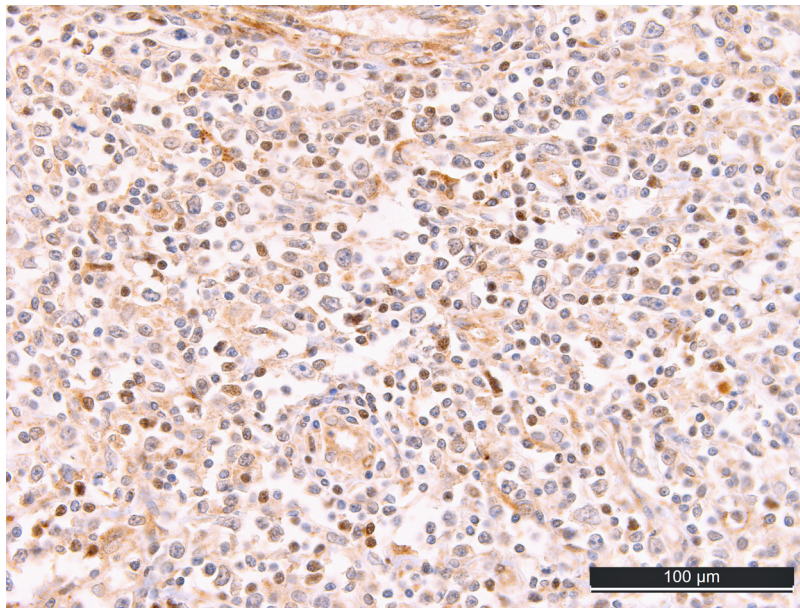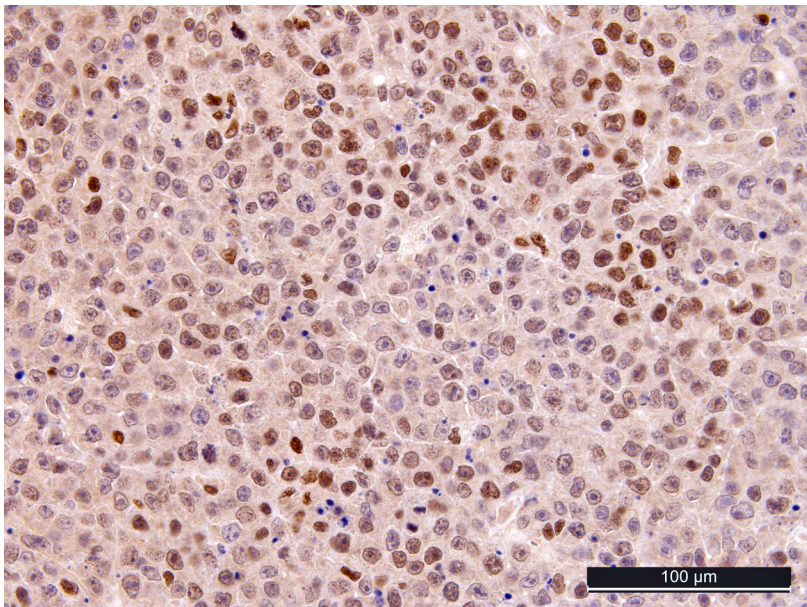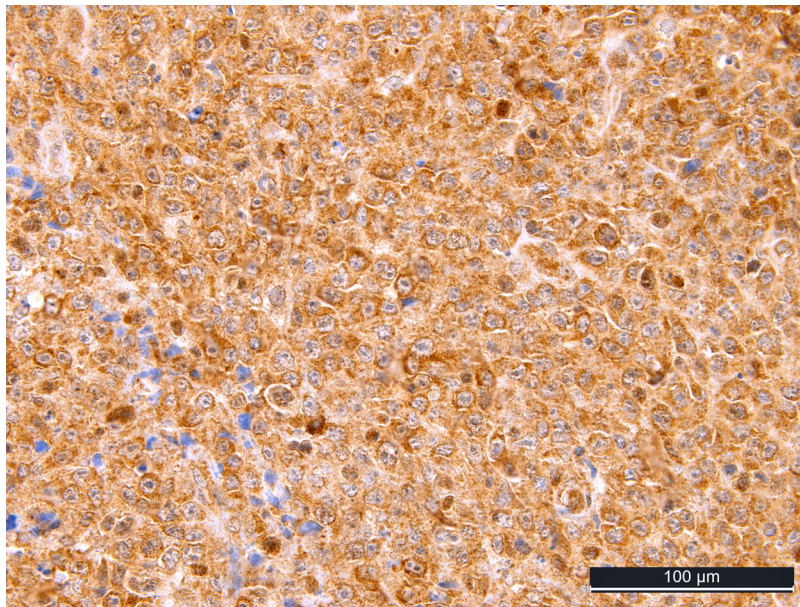

Supplement: Supplementary file 6 — Source Data for Figure 6 [file EMMM-11-e10576-s004.pdf]

**F**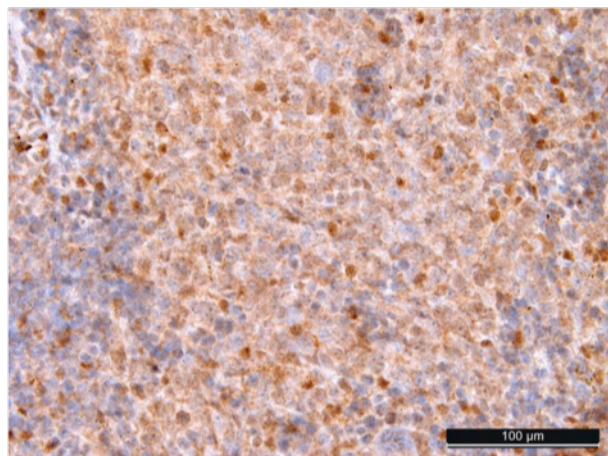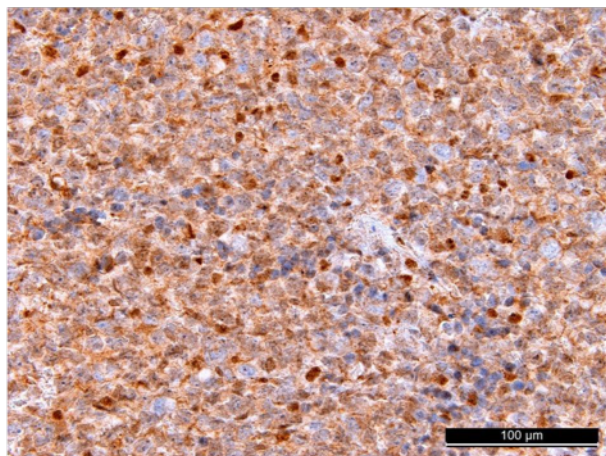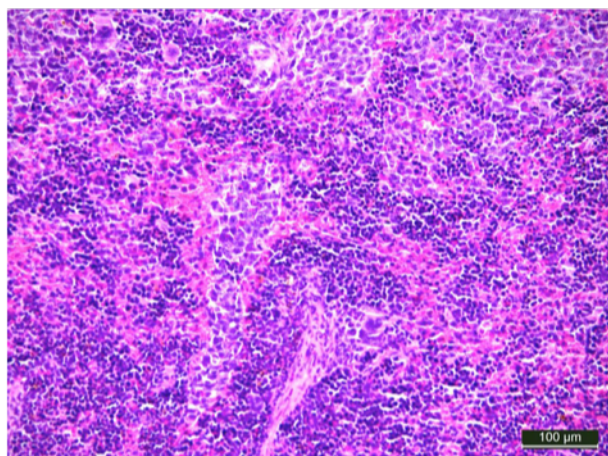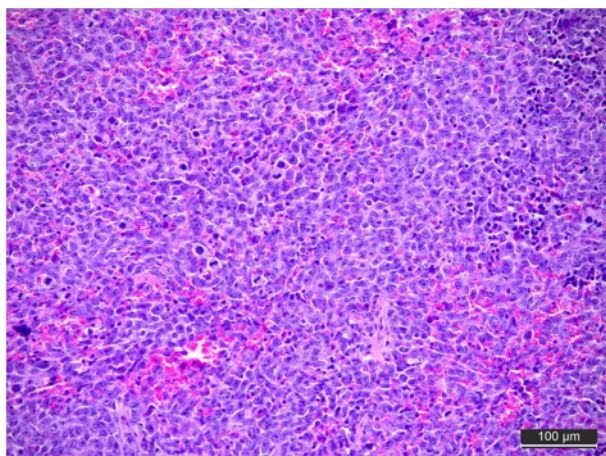

Supplement: Supplementary file 7 — Source Data for Figure 8 [file EMMM-11-e10576-s005.pdf]
